# New Frontier on Antimicrobial Therapy: Long-Acting Lipoglycopeptides

**DOI:** 10.3390/pathogens13030189

**Published:** 2024-02-20

**Authors:** Valentina Siciliano, Flavio Sangiorgi, Pierluigi Del Vecchio, Layla Vahedi, Maya Manuela Gross, Angela Saviano, Veronica Ojetti

**Affiliations:** 1 Dipartimento di Scienze di Laboratorio e Infettivologiche, Fondazione Policlinico Universitario Agostino Gemelli IRCCS, 00168 Rome, Italy; 2 Dipartimento di Sicurezza e Bioetica, Università Cattolica del Sacro Cuore, 00168 Rome, Italy; flavio.sangiorgi@guest.policlinicogemelli.it (F.S.); pierluigidelvecchio92@gmail.com (P.D.V.); laylavahedi9@gmail.com (L.V.); maya.gross@hotmail.com (M.M.G.); 3 Dipartimento di Emergenza Urgenza, Fondazione Policlinico Universitario Agostino Gemelli IRCCS, 00168 Rome, Italy; angela.saviano@policlinicogemelli.it (A.S.); veronica.ojetti@policlinicogemelli.it (V.O.)

**Keywords:** long-acting lipoglycopeptides, dalbavancin, oritavancin

## Abstract

Long-acting lipoglycopeptides (LGPs), such as dalbavancin and oritavancin, are semisynthetic antibiotics known for their strong effectiveness against a wide array of Gram-positive bacteria. This includes *Staphylococcus aureus*, both methicillin-sensitive (MSSA) and methicillin-resistant (MRSA) strains, *coagulase-negative Staphylococci* (CoNS), streptococci, and vancomycin-sensitive *Enterococcus faecalis*. A literature search was conducted on PubMed and on ClinicalTrials.gov to identify articles published until July 2023 investigating the use of oritavancin and dalbavancin in clinical practice. The review included case reports, case series, observational studies, and clinical studies. Although more consistent data are needed, LGPs seem to be a good alternative that may provide a quicker hospital discharge and reduce long-term intravenous access and therapy. This is attributed to their unique pharmacologic and pharmacokinetic characteristics. More quality data (i.e., number of patients treated with clinical success) are needed before clinicians may use these therapies more widely.

## 1. Introduction

The long-acting lipoglycopeptides (LGPs) are a group of semisynthetic antibiotics that include dalbavancin and oritavancin, which have potent activity against a broad spectrum of Gram-positive pathogens, including both methicillin-sensitive (MSSA) and methicillin-resistant (MRSA) *Staphylococcus aureus*, *coagulase-negative Staphylococci* (CoNS), streptococci, and vancomycin-susceptible *Enterococcus faecalis* [1,2].Their bactericidal activity is mediated by the inhibition of cell-wall biosynthesis, binding to the D-alanyl-D-alanine terminus of the lipid II bacterial cell-wall precursor, and preventing cross-linking of the peptidoglycan layer [3]. Furthermore, oritavancin appears to possess supplementary mechanisms of action. Specifically, it inhibits the transglycosylation process within the cell wall, resulting in a disruption of cell-wall integrity. This disruption leads to depolarization, permeabilization, and rapid bacterial death [4]. This group of antibiotics has many characteristics in common with glycopeptides (e.g., vancomycin, teicoplanin) including the spectrum of antimicrobial action, but at the same time presents notable differences, such as a greater potency of action on many target pathogens, and a longer half-life, which allows it to be administered less frequently [1,2].

In particular, dalbavancin has a long terminal half-life ranging from 149 to 250 h in human subjects [5]. Oritavancin is a semisynthetic analogue of vancomycin, whereas dalbavancin is obtained by a fermentation process using the actinomycete Nonomuraea sp. followed by structural modifications; specifically, the increased potency and prolonged half-life result from the addition of an extended lipophilic side chain, while the increased antistaphylococcal activity results from the addition of an amidated carboxyl side group [6]. Oritavancin is structurally similar to vancomycin, however, with structural variations that are responsible for its greater antimicrobial potency on Gram-positive bacteria even with *van*A- and *van*B-mediated resistance. It has a half-life ranging from 200 to 300 h [2]. Currently, these antibiotics are approved by the Food and Drug Administration (FDA) only for the treatment of acute bacterial skin and skin structure infections (ABSSSIs). However, thanks to their powerful antimicrobial activity and long terminal half-life, they seem to perform a promising activity regarding various types of more severe and profound infections, such as bone and joint infections (BJIs), implanted-associated infections (IAIs), infective endocarditis (IE), and bloodstream infections (BSIs). In particular, their longer duration of action and consequently their less frequent administration translate into numerous advantages, including earlier hospital discharge, shorter residence time of vascular catheters, a reduction in the probability of infection of the same, and greater compliance by the patient with less impact on his daily life. Due to their lack of oral absorption, these antibiotics are typically administered intravenously (IV). They do not require dosage adjustment in the elderly population, in patients with mild or moderate renal impairment (ClCr > 30 mL/min), or even in patients on regularly scheduled hemodialysis, (e.g., three times a week). Dosage adjustment is necessary only in cases of severe renal impairment (ClCr < 30 mL/min) [1,2]. The pharmacokinetics (PKs) of dalbavancin are linear, therefore increasing the dose increases its plasma concentration. Approximately 93% of dalbavancin is bound to human plasma proteins, in particular to albumin, and 7% exists in an unbound form. These percentages remain substantially unchanged depending on the concentration of the drug in the plasma and on liver and kidney function. The pharmacokinetics of oritavancin is also linear and is approximately 85% bound to human plasma proteins [5,6]. Lipoglycopeptides, while showing a similar spectrum of action to glycopeptides, exert greater potency than these. Against MRSA, dalbavancin has been shown to have a potency 16 times greater than daptomycin and 32 times greater than vancomycin and linezolid. It is also the most powerful agent on the CoNS [5]. Against MRSA, oritavancin showed 8-fold greater potency than daptomycin and 16- to 32-fold greater potency than vancomycin and linezolid [2]. However, dalbavancin is not active against enterococci with glycopeptide resistance mediated by *van*A genes and is only partially active against *van*B strains [7]. Oritavancin, due to its additional mechanisms of action, shows potent in vitro activity against both *van*A and *van*B vancomycin-resistant enterococci, a feature that makes it unique among lipoglycopeptides. Moreover, it has synergistic activity against VRE when administered together with beta-lactams [4,8,9]. As mentioned, dalbavancin and oritavancin show an extremely long half-life (7–14 days), thus being able to exert a more prolonged selective pressure on exposed bacteria. Resistance to LPGs is not commonly observed in clinical practice. To date, the precise mechanisms underlying dalbavancin resistance remain poorly understood. Most VRE strains are known to exhibit a resistance phenotype to this antibiotic. As for oritavancin, although it is active against both *van*A and *van*B enterococci, it has been seen that the presence of multiple copies of the *van* gene cluster leads to a low level of resistance to oritavancin, with an increase in the Minimum Inhibitory Concentration (MIC) of about three times [10]. Furthermore, the *van*Z gene also contributes to oritavancin and teicoplanin resistance through unclear mechanisms [11]. From this point of view, it is clear that antibiotic resistance and the mechanisms that mediate it within the group of LGPs are topics to be further investigated and better understood.

The profile of adverse effects reported is mostly mild, including nausea, headache, reactions at the site of infusion, rash, phlebitis, and less frequently also *Clostridioides difficile* colitis, liver toxicity, and reversible acute renal failure.

LGPs may prolong the prothrombin time (PT) and international normalized ratio (INR) for up to 12 h. There are no available studies on pregnant or lactating women.

This narrative review aims to summarize the current evidence about the use of LGPs in BSIs, IE, and PJIs.

## 2. Materials and Methods

A literature search was conducted on PubMed and ClinicalTrials.gov website to identify articles investigating the use of oritavancin and dalbavancin in clinical practices that were published until July 2023. We focused the search on BSIs, IE, BJIs, and IAIs in adults. For this review, the focused search was performed on the PubMed library database using the following keywords: “dalbavancin” or “oritavancin” in pair with the specific origin of infection (“bacterial bloodstream infection”, “infective endocarditis”, “bone and joint infection”, and “implanted-associated infections”). Case reports, case series, observational studies, and clinical studies were included. The search was limited to results in the English language and results from languages other than English were excluded.

## 3. Results

### 3.1. Dalbavancin

#### 3.1.1. BSI and IE

We summarize in Table 1 the findings of reports, including endocarditis cases, BSIs, and catheter-related bloodstream infections (CRBSIs), with documented clinical outcomes.

As shown, regarding IE, a total of 194 patients have been treated with dalbavancin, with a broad variety of IE (native/prosthetic valve, cardiac-device related), dosing and duration of therapy, and pathogens that cause infections. The majority of the published work with dalbavancin regards infections due to staphylococci, enterococci, and streptococci.

Dalbavancin was mainly used as a second-line agent in the last weeks of therapy for suppressive therapy or, in a few cases, as rescue therapy if there was a failure to clear the bloodstream with a first-line agent. Clinical and microbiological success ranged from 57% to 100%, with an overall success rate of 82%. The most common dosing regimen is a 1000 to 1500 mg first loading dose, followed by 375 to 1500 mg weekly, with a wide variation in the dosage.

In all the studies, dalbavancin appears to be well tolerated in IE patients, with few adverse events that are non-severe.

Regarding bloodstream infection (BSI) and CRBSI, we collected, as shown in Table 2, 79 patients that have been treated with dalbavancin as monotherapy, combination therapy, first-line therapy, or consolidation therapy in the last 2 years. As already underlined, the majority of the published papers regard infections due to staphylococci, enterococci, streptococci, and, in a few cases, *Corynebacterium* spp. [12]. Clinical and microbiological success range from 86% to 100%, with an overall success rate of 93.7%. The most common regimen in this case is a 1500 mg first loading dose followed by 1125 to 1500 mg weekly, with a different duration as appropriate. Here too, dalbavancin seems to be well tolerated in all the studies.

A recent pilot study also assessed the efficacy and safety of a single-step treatment strategy for CRBSI, combining a single dalbavancin administration (1500 mg) and catheter removal with a quick discharge from the hospital. They enrolled 16 patients with confirmed Gram-positive (mostly staphylococci) CRBSIs. Most of the infected devices were central venous catheters (CVCs) and peripherally inserted central catheters (PICCs). The diagnoses at hospital admission were mostly sepsis and respiratory failure. Clinical success at the end of the 30-day follow-up period was 100% with a good safety profile. Not one of the patients was readmitted within 30 days of the first diagnosis. This study may indicate that this strategy could be highly effective and cost-effective for patients with Gram-positive CRBSI [13].

#### 3.1.2. BJI and IAI

Effective management of osteoarticular infections, including those involving prosthetic joints, necessitates a well-balanced approach that combines optimal surgical and medical interventions to enhance the likelihood of successful treatment outcomes [14]. Challenges in achieving therapeutic success include issues related to patient tolerance, difficulties in maintaining adherence throughout the extended treatment duration needed for bone and joint infections, the emergence of bacterial multidrug resistance, and the sometimes-invasive nature of intravenous antibiotic administration requiring prolonged hospitalization [15].

Dalbavancin tackles several of these obstacles. With its extended half-life, it can be conveniently administered through a simple injection, eliminating the requirement for a central line and avoiding prolonged hospital stays [15]. Moreover, it exhibits efficacy against staphylococci and other Gram-positive pathogens and is generally well-tolerated by patients [14].

Various retrospective studies, clinical reports, and case series have documented the utilization of dalbavancin in the management of osteomyelitis, septic arthritis, prosthetic joint infections (PJIs), and other bone and joint infections.

Collectively, the articles summarized in Table 2 encompass a total of 666 patients who received dalbavancin for either consolidation therapy following a successful initial regimen or as salvage therapy. The overall clinical success rate in these studies stood at 74%, with success rates ranging from 50% to 100%. This success rate exhibited considerable variation depending on the type of infection (between implanted-associated infection and bone and joint infection), the specific indication for dalbavancin (salvage therapy or consolidation therapy), and the surgical interventions performed.

Seven out of the seventeen studies included in Table 3 did not report any adverse events. Of those that did report adverse events, they were typically mild and occurred in less than 5% of patients, the most common of which were nausea and vomiting (<2%), infusion-related reactions (<2%), and acute kidney injury (<2%).

A major hurdle observed in the clinical use of dalbavancin is the lack of uniformity in dosing regimens, which is noticeable even within the same set of cases. Furthermore, there is a notable absence of comprehensive data, including the rate of success by infection location or type, causative agents, primary and secondary outcome measures, and definitions.

### 3.2. Oritavancin

#### 3.2.1. BSI and IE

Oritavancin, as highlighted above, is only approved by the FDA for the treatment of ABSSSIs.

The first study about the use of oritavancin in the treatment of BSIs was a phase 2 randomized study in which patients with uncomplicated *S. aureus* bacteremia (n = 86) received either oritavancin at 5 to 10 mg/kg of body weight or standard-of-care (SOC) therapy with a beta-lactam or vancomycin (for MSSA or MRSA, respectively). For 55 patients treated with oritavancin, clinical and microbiological success were observed in 47 (85%) and 45 (78%) patients, respectively [16].

The most recent is a monocentric study where all 72 patients included in the BSI, received SOC therapy before oritavancin with the aim of early discharge. The pathogens more frequently isolated were *S. aureus* (mostly MSSA), *Streptococcus* spp., and *Enterococcus* spp. The clinical and microbiological failures were 14% and 5%, respectively [17].

Other observational studies and case reports are smaller (1 to a maximum of 7 patients each), summarized in Table 3. They confirmed the use of 1200 mg of oritavancin for BSIs or for endocarditis (only six patients) as a follow-up regimen after a previous treatment with other antimicrobials.

#### 3.2.2. BJI and IAI

A similar use of oritavancin as a consolidation regimen is reported in case reports, and retrospective studies of BJIs and IAIs are reported in Table 1. The isolates are the same as those reported for bacteremia, such as staphylococci and enterococci. In this scenario, oritavancin was often prescribed with a 1200-mg loading dose followed by 800 to 1200 mg weekly (only a few reports reported a single dose of antibiotic).

In the largest study by Van Hise et al., 134 patients with acute osteomyelitis were treated with oritavancin 1200 mg and then 800 mg weekly for 4 or 5 weeks. In total, 128 patients had positive cultures, and 92 of them (72%) had MRSA. At 7–10 days from the last dose of oritavancin 118 patients (88%) achieved clinical success, and 13 patients (9.7%) had a relapse or persistent infection at 6 months of follow-up [18].

Other case reports and series are smaller (1 to a maximum of 25 patients) with a similar rate of clinical success at a mean follow-up of up to 6 months.

The data reported are so heterogeneous in terms of microbiological isolates, treatment patterns, and types of infection that they represent only a starting point for more structured, large-scale studies about the use of oritavancin in bone and joint infections.

**Table 1:** Main characteristics of the studies about the use of dalbavancin in bloodstream infections and endocarditis included in the review (arranged by publication year).

Authors (Year) [Bibliography Reference]	N of Patients	Infection(s) (n)	Pathogens (n)	Most Frequent Dosages	Duration/n of Doses	Success ^a^, n (%)	Adverse Event(s) (n)
Tobudic et al., (2018) [19]	27	NVE (15), PVE (7), CDE (5)	*S. aureus* (9), CoNS (7), *E. faecalis* (4), other (9)	1500 mg once then 1000 mg every 2 wk or 1000 mg once then 500 mg weekly	1–30 weeks	25 (93)	Nausea (1), RCI (1)
Bouza et al. (2018) [20]	7	EI (7)	*S. aureus* (1), CoNS (2), *Enterococcus* spp (2), other (2)	1000 mg once then 500 mg weekly	3 doses	6 (86)	Rash (2), tachycardia (2), reversible kidney injury (2), nausea (1), rectal bleeding (1)
Hidalgo-Tenorio et al., (2019) [21]	34	NVE (11), PVE (15), CDE (8)	*S. aureus* (10), CoNS (15), *E. faecalis* (3), other (7)	1000–1500 mg once then 500 mg at day 8	14 days	33 (97)	Fever (1), renal failure (1)
Bryson-Cahn et al., (2019) [22]	9	NVE (9)	*S. aureus* (9)	1000–1500 mg once then 500 mg at day 7	2 doses	9 (100)	N/R
Wunsch et al., (2019) [23]	25	NVE (15), PVE (6), CDE (4)	Not specified	1000 mg once then 500 mg weekly or 1500 mg once or 1500 mg twice	1–3 doses	23 (92)	Dyspnea (1), IRR (1), fatigue and vertigo (1)
Dinh et al., (2019) [24]	19	NVE (9), PVE (10)	Not specified	1500 mg once or 1500 mg once then 1000-1500 mg at day 7 or 14	1–2 doses	13 (68)	Hypersensitivity (2), headache (1), eosinophilia (1), phlebitis (1)
Bork et al., (2019) [25]	7	EI (7)	Not specified	Not specified	4 doses	4 (57)	Acute kidney injury (2), rash and pruritus (1)
Veve et al., (2020) [26]	12	EI (12)	Not specified	1500 mg once or 1500 mg once then 1500 mg at day 7 or day 14	1–2 doses	N/R	Catheter infection (1), hypersensitivity (1)
Evins et al., (2022) [27]	23	BSI	MSSA (7), MRSA (6), *S. epidermidis* (2), *E. faecalis* (2), streptococci (3)	1125–1500 mg once or 1500 mg weekly for two doses	1–2 doses	23 (100)	N/R
Tuan et al., (2022) [28]	9	Not specified	Staphylococci, streptococci, enterococci, *Corynebacterium* spp.	1500 mg once or 1500 mg at day 1 and 8	1–2 doses	9 (100)	Hepatotoxicity (1)
Taylor et al., (2022) [12]	18	BSI (7), EI (11)	MRSA, MSSA, CoNS, *Corynebacterium* spp, *E. faecalis*	1500 mg every 14 days	1–4 doses	9 (83)	N/R
Lueking et al., (2023) [29]	27	BSI (23), EI (4)	MRSA, MSSA, CoNS, *Enterococcus* spp, streptococci	1500 mg once or 1125 mg once or 1500 mg at day 1 and 8	1–2 doses	23 (85.1)	*Clostridioides* difficile colitis (1), substernal chest pain during infusion (1).
Teigell-Munoz et al., (2023) [30]	1	NVE (1)	*E. faecalis*	1000 mg once then 500 mg at day 8	1 dose	1 (100)	N/R
Ruiz-Sancho et al., (2023) [31]	6	PVE (6)	*E. faecium* (1), MSSA (2), *S. epidermidis* (1), *S. gallolyticus* (1), other (1)	1000–1500 mg once then 500–1500 mg at day 7 or 14	2 doses	5 (83)	Asthenia (1), liver and kidney injury (1).
Ioannou et al., (2023) [32]	19	6 BSI, 13 EI	*S. aureus*, Enterococci, streptococci, CoNS	1500 mg every two weeks	N/R	12 (94.7)	N/R
Mansoor et al., (2023) [33]	10	LVAD infection	*C. striatum* (7), MRSE (2), *C. amycolatum* (1)	1000–1500 mg every two weeks, 375–500 mg every week	N/R	6(60)	N/R
Venturini et al., (2023) [13]	16	CRBSI (16)	MSSA (5), MRSA (1), MRSE (3), MSSE (3), *S. capitis* (3), *E. faecalis* (1)	1500 mg once	1 dose	16 (100)	None

Abbreviations: NVE (native valve endocarditis); PVE (prosthetic valve endocarditis); CDE (cardiac device endocarditis); CoNS (Coagulase-negative staphylococci); EI (infective endocarditis); N/R (not reported); IRR (infusion-related reactions); BSI (bloodstream infection); MSSA (methicillin-susceptible *S. aureus*); MRSA (methicillin-resistant *S. aureus*), LVAD (left ventricular assist device); MRSE (methicillin-resistant *S. epidermidis*); MSSE (methicillin-susceptible *S. epidermidis*); CRBSI (catheter-related bloodstream infection). ^a^: Definitions of clinical success were heterogeneous across the studies. For details, refer to the individual publication.

**Table 2:** Main characteristics of the studies about the use of dalbavancin in implant-associated infection and bone and joint infections included in the review (arranged by publication year).

Authors (Year) [Bibliography Reference]	N of Patients	Infection(s) (n)	Pathogens (n)	Most Frequent Dosages	Duration/n of Doses	Success ^a^, n (%)	Adverse Event(s) (n)
Bouza et al. (2018) [20]	33	IAI (20), BJI (13)	*S. aureus* (9), CoNS (16), *Enterococcus* spp (3), other (6)	1000 mg once then 500 mg weekly	3 doses	28 (85)	Rash (2), tachycardia (2), reversible kidney injury (2), nausea (1), rectal bleeding (1), candidiasis (1)
Rappo et al., (2019) [34]	67	BJI (67)	*S. aureus* (42), CoNS (14), *Enterococcus* spp (8), other (33)	1500 mg weekly × 2	2 doses	65 (97)	IRR (1)
Morata et al., (2019) [35]	64	IAI (45), BJI (19)	*S. aureus* (14), CoNS (33), *Enterococcus* spp (9), other (22)	1000 mg once then 500 mg weekly	2–5 doses	45 (70)	Gastrointenstinal symptoms (3), rash (1), phlebitis (1), asthenia (1), reversible kidney injury (1)
Almangour et al., (2019) [36]	31	BJI (31)	*S. aureus* (27), CoNS (1), other (6)	1000 mg once then 500 mg weekly or 1500 mg weekly × 2	2–3 doses	28 (90)	None
Tobudic et al., (2019) [37]	46	IAI (8), BJI (38)	Not specified	1500 mg once then 1000 mg every 2 wk or 1000 mg once then 500 mg weekly or 1500 mg at day 1 and day 8	2–32 doses	30 (65)	Nausea (1), exanthema (2), hyperglycemia (1)
Dinh et al., (2019) [24]	48	BJI (48)	Not specified	1500 mg once or 1500 mg once then 1500 mg every 2 wk	110 doses	35 (73)	Hypersensitivity (2), headache (1), eosinophilia (1), phlebitis (1)
Wunsch et al., (2019) [23]	62	IAI (32), BJI (30)	Not specified	1000 mg once then 500 mg weekly or 1500 mg once or 1500 mg twice	1–3 doses	58 (94)	Dyspnea (1), IRR (1), fatigue and vertigo (1)
Buzon-Martin et al., (2019) [38]	16	IAI (16)	*S. aureus* (6), CoNS (7), *Enterococcus* spp (6),	1500 mg once then 500 mg on day 7 and every 2 wk	6–12 wk	11 (69)	Leukopenia (1), rash (1)
Bork et al., (2019) [25]	15	Not specified	Not specified	Not specified	4 doses	7 (47)	AKI (2), rash (1)
Veve et al., (2020) [26]	49	Not specified	Not specified	1500 mg once or 1500 mg once then 1500 mg at day 7 or day 14	1–2 doses	N/R	Catheter infection (1), hypersensitivity (1)
Matt et al., (2021) [39]	17	IAI (17)	*S. aureus* (10), CoNS (10), *E. faecalis* (1), other (5)	1500 mg once or 1500 mg weekly ×2	1–2 doses	8 (47)	None
Cojutti et al., (2021) [40]	15	IAI (11), BJI (4)	*S. aureus* (5), CoNS (9), *E. faecalis* (1),	1500 mg weekly × 2	2 doses	12 (80)	None
Tuan et al., (2022) [28]	23	BJI (21), SA (2)	Staphylococci, streptococci, enterococci, *Corynebacterium* spp.	1500 mg once or 1500 mg at day 1 and 8	1–2 doses	21 (91.3)	Hepatotoxicity (1)
Taylor et al., (2022) [12]	30	IAI (4), BJI (26)	MSSA, MRSA, CoNS, *Corynebacterium* spp, *E. faecalis*	1500 mg every 14 days	1–4 doses	26 (87)	N/R
Cain et al., (2022) [41]	42	BJI (42)	*S. aureus* (23), other (19)	1500 mg once	1 dose	33 (78.6)	Nausea, IRR
Mazzitelli et al. (2022) [42]	15	Spondylodiscitis	MRSA	1500 mg at day 1 and 8, then 1500 mg every 28–35 days	3–14 doses	14 (93.3)	None
Lueking et al., (2023) [29]	20	BJI 16), SA (4)	MSSA, MRSA, CoNS, *Enterococcus* spp, streptococci	1500 mg once or 1125 mg once or 1500 mg at day 1 and 8	1–2 doses	18 (90)	Clostridioides difficile colitis (1), substernal chest pain during infusion (1).
Soderquist et al., (2023) [43]	1	IAI (1)	*Corynebacterium striatum*	1000 mg once then 500 mg every week	12 weeks	1(100)	N/R
Ruiz-Sancho et al., (2023) [31]	2	IAI (2)	*E. faecium* (1), *S. epidermidis* (1)	1000 mg once then 500 mg every week	N/R	1 (50)	None
Ioannou et al., (2023) [32]	55	IAI, SA (not specified)	*S. aureus*, Enterococci, streptococci, CoNS	1500 mg every two weeks	N/R	42 (76)	N/R
Doub et al. (2023) [44]	15	IAI (15)	*C.striatum* (2), MSSA (3), MRSA (3), CoNS (4), other (4)	1500 mg at day 1 and 8	2 doses	13 (86.6)	N/R

Abbreviations: IAI (implanted-associated infection); BJI (bone and joint infection); CoNS (Coagulase-negative staphylococci); WK (weeks); AKI (Acute kidney injury); N/R (not reported); SA (septic arthritis); MSSA (methicillin-susceptible *S. aureus*); MRSA (methicillin-resistant *S. aureus*). ^a^: Definitions of clinical success were heterogeneous across the studies. For details, refer to the individual publication.

**Table 3:** Main characteristics of the studies about the use of oritavancin included in the review (arranged by publication year).

Authors (Year) [Bibliography Reference]	*N* of Patients	Infection(s) (*n*)	Pathogens (*n)*	Most Frequent Dosages	Duration/n of Doses	Success ^a^, *n* (%)	Adverse Event(s) (*n*)
Bhavnani et al. (2006) [16]	55	Bacteremia (55)	*S. aureus* (55)	5–10 mg/kg/day	10–14 days	45 (78)	N/R
Johnson et al., (2015) [45]	1	PVE (1)	*E. faecium* VRE (1)	1200 mg weekly every 48 h × 3 doses, then 1200 mg × 6 wk, then 1200 mg biweekly	14 doses	1 (100)	Anorexia, nausea (1)
Stewart et al., (2017) [46]	8	Bacteremia (6), NVE (1) and bursitis (1)	MSSA (4), CoNS (1), *Enterococcus* spp (1), *S. agalactiae* (1)	1200 mg	1 dose	5 (62.5)	Hearing loss (1)
Foster et al., (2017) [47]	1	IAI (1)	*E. faecium* VRE (1)	1200 mg weekly	6 doses	1 (100)	None
Delaportas et al., (2017) [48]	1	Acute osteomyelitis (1)	MSSA (1)	1200 mg weekly	7 doses	1(100)	None
Ruggero et al., (2018) [49]	1	Acute osteomyelitis (1)	MRSA (1)	1200 mg every 2–4 wk	5 doses	1 (100)	N/R
Schulz et al., (2018) [50]	5	Bacteremia (1); acute and chronic osteomyelitis, septic arthritis, discitis (4)	MSSA (1), *E.faecium* VRE (1), other (3)	1200 mg then 800 mg weekly	2–8 doses	2 (40)	Anemia and leukopenia (1)
Datta et al., (2018) [51]	3	Bacteremia (3)	MRSA (1), *S. gallolyticus* (1), *Granulicatella adiacens* (1)	1200 mg	1 dose	3 (100)	N/R
Redell et al., (2019) [52]	32	Bacteremia (7); acute and chronic osteomyelitis, septic arthritis, IAI (25)	MRSA (2), MSSA (1), *S. epidermidis* (1), other (28)	1200 mg once or every 6–14 days	1–10 doses	26 (81.2%)	Not specified (29)
Dahesh et al., (2019) [53]	1	IAI (1)	*E.faecium* VRE (1)	1200 mg × 2 wk then 800 mg weekly	10 doses	1 (100)	N/R
Chastain and Davis, (2019) [54]	9	Chronic osteomyelitis	MRSA (5), other (4)	1200 mg once then 1200 mg every 13–52 days	2–6 doses	9 (100)	None
Brownell et al., (2020) [55]	20	Endocarditis (4); osteomyelitis, diabetic foot, IAI (16)	Not specified	1200 mg once then 800–1200 mg weekly	N/R	20 (100)	Not specified (3)
Van Hise et al., (2020) [18]	134	Acute osteomyelitis (134)	MSSA (35), MRSA (108), VISA (2), *E. faecium* VRE (7)	1200 mg once then 800 mg weekly	4–5 doses	118 (88.1)	Hypoglycemia (3), tachycardia (2)
Texidor et al., (2023) [17]	72	Bacteremia (72)	Polymicrobial (20), MRSA (12), MSSA (37), streptococci (19), *E. faecium* (3), *E. faecium* VRE (4), CoNS (6), other (4)	800–1200 mg once, followed by 800–1200 mg	1–2 doses	52 (81.2%), N/R for 8 patients	AKI (3), IRR (2)

Abbreviations: N/R (not reported); PVE (prosthetic valve endocarditis); VRE (vancomycin resistant enterococci); WK (week); NVE (native valve endocarditis); MSSA (methicillin-susceptible *S. aureus*); CoNS (Coagulase-negative staphylococci); IAI (implant-associated infection); MRSA (methicillin-resistant *S. aureus*), VISA (vancomycin-intermediate *S. aureus*); AKI (acute kidney injury), IRR (infusion-related reactions).^a^: Definitions of clinical success were heterogeneous across the studies. For details, refer to the individual publication.

## 4. Conclusions

In summary, the long-acting lipoglycopeptides (LGPs), represented by dalbavancin and oritavancin, showcase remarkable efficacy against a broad spectrum of Gram-positive pathogens, including methicillin-sensitive and methicillin-resistant Staphylococcus aureus, coagulase-negative Staphylococci, streptococci, and vancomycin-susceptible Enterococcus faecalis. Their bactericidal activity, mediated by inhibiting cell-wall biosynthesis, makes them potent agents in combating infections.

The distinctive features of these antibiotics, such as an extended half-life, allow for less frequent administration, offering advantages like earlier hospital discharge, reduced catheter residence time, and improved patient compliance. Despite the current FDA approval for the treatment of acute bacterial skin and skin structure infections, the robust antimicrobial activity and prolonged half-life suggest their potential efficacy in more severe infections, including bone and joint infections, implanted-associated infections, infective endocarditis, and bloodstream infections.

Dalbavancin, with a half-life ranging from 149 to 250 h, and oritavancin, with a half-life of 200–300 h, exhibit potent activity against resistant strains, although dalbavancin may face limitations against enterococci with *van*A-mediated resistance. Both antibiotics demonstrate a prolonged selective pressure on bacteria due to their extended half-life, yet resistance remains uncommon in clinical practice.

The reported adverse effects, predominantly mild, include nausea, headache, infusion site reactions, rash, and less frequently, Clostridioides difficile colitis, liver toxicity, and reversible acute renal failure. Notably, LGPs may influence prothrombin time and international normalized ratio for up to 12 h.

In our comprehensive literature review, dalbavancin emerges as a promising therapeutic option in infective endocarditis, bloodstream infections, and implant-associated infections, showcasing high clinical and microbiological success rates. Oritavancin, though primarily FDA-approved for skin infections, demonstrates potential in treating bacteremia and bone/joint infections. However, the heterogeneity in dosing regimens and limited structured data highlight the need for further large-scale studies to establish standardized guidelines for optimal use.

In conclusion, the evidence presented in this narrative review underscores the promising role of LGPs in the management of severe Gram-positive infections. As we move forward, additional research is imperative to refine dosing regimens, understand resistance mechanisms, and expand the scope of LGPs in diverse clinical scenarios. The reviewed studies collectively contribute to building a foundation for informed decision-making in the clinical use of dalbavancin and oritavancin, offering potential avenues for improved patient outcomes in challenging infectious scenarios.
